# EgyGene GelAnalyzer4: a powerful image analysis software for one-dimensional gel electrophoresis

**DOI:** 10.1186/s43141-020-00114-x

**Published:** 2021-01-25

**Authors:** N. E. Ahmed

**Affiliations:** Genetics Department, Faculty of Agriculture, Ain Shams University, Cairo, Egypt

**Keywords:** EgyGene, GelAnalyzer, Image analysis, Gel image analysis, Image enhancement, Merging data, Molecular markers, Phylogenetic tree, Population parameters

## Abstract

**Background:**

Gel image analysis is a cornerstone in electrophoresis-based experiments. Nature of the experiment determines the target of the analysis. Implemented features within different software for gel images and their outputs are variable. EgyGene GelAnalyzer4 software aimed to make a powerful, easy, and almost all-in-one software for gel image analysis.

**Results:**

EgyGene GelAnalyzer4 is a software for analyzing the gel images. The program consists of six main parts: gel image enhancement, image analysis, data merging, molecular markers detection, phylogenetic tree prediction, and population parameters estimation. Firstly, the gel image enhancement part enhances the gel image colors and clarity. Then, the image is analyzed in the second part using both automatic and manual detection of lanes and bands. The third part, data merging, is for merging several analyses for different samples/gels together to produce one table comprises the entire experiment. The fourth section, markers detection, collects several pre-analyzed data for the same samples to detect their molecular markers based on some user-defined criteria. The fifth part uses the outputs of the previous parts to draw the phylogenetic tree between samples. The program constructs a phylogenetic tree using unweighted pair group method with arithmetic mean (UPGMA) method. Fourteen different equations are used to calculate the similarity percentages: Anderberg, Czekanowski, Dice, Faith, Gower and Legendre, Jaccard, Nei and Li, Rogers and Tanimoto, Russel and Rao, simple matching, Sokal and Michener, Sokal and Sneath 1, Sokal and Sneath 2, and Sorensen. The last section calculates the population parameters for the pre-analyzed images before. EgyGene GelAnalyzer4 is designed and programed using Microsoft Visual Studio 2012 Express version, based on the Dot Net FrameWork 4.5 edition, whereas its icons are created using the freeware Greenfish Icon Editor Pro 3.2.5.1.

**Conclusions:**

EgyGene GelAnalyzer4 is a simple, fast, and user-friendly software with six novel features: changing the order of lanes within the gel image, splitting the detecting lane into two lanes, merging of several detected lanes into just one, splitting a single band into two bands, merging two or more bands to be a single band, and comparing data of several analyzed images with a big number of samples and merging it together in a single table and detection of molecular markers between samples. In addition to the basic features and outputs of other image analysis software such as calculation of relative mobility values, molecular weights/sizes estimation, DNA/RNA/protein quantity within bands, table of present/absent of bands, polymorphism percentage, similarity percentages and phylogeny, population parameters, enhancing the image colors and clarity, and merging data for several gels. In addition, writing names of samples and the detected markers on the image. EgyGene GelAnalyzer4 provides many several features, which may be not available together in any other alternative.

## Background

Analysis of gel image is a daily task in molecular biology laboratories. The goal of analyzing the gel image depends on the experiment. In gene expression studies, the target is mainly to measure the differences in band intensity between the samples under the studied conditions [1, 2, 17]. In fingerprinting studies, the main goal is to observe the differences in the banding pattern between samples [6, 15]. In bulked segregate analysis, researchers are focusing on the detection of molecular markers [1, 3, 19]. Measuring of gene flow experiments needs the population parameters like gene/genotypic frequency, heterozygosity, and gene diversity [6, 9, 13]. Gel image software varies in their outputs [10]. For example, the freeware JAVA-based GelAnalyzer2010a does not include phylogenetic analysis while the freeware PyElph1.4 provide it [10]. However, GelAnalyzer2010a determines the quantity of protein in the band whereas *PyElph1.4* does not. It is hard (if it is possible) to find one gel analysis software produces all the outputs you need [8]. For example, the powerful freeware *PyElph1.4* makes a comparison between bands across all lanes but it is not determining molecular markers for specific lane(s) [8]. The main target of EgyGene GelAnalyzer4 program is to make an all-in-one software for gel analysis. We aimed to make it simple to use, fast, user-friendly, feature-enriched, integrative, attractive, doing the most of the job with the least mouse clicks, and produces all the outputs the researchers need regardless their experiment type.

## Methods

### Programing language

EgyGene GelAnalyzer4 is designed and programed using Microsoft Visual Studio 2012 Express; a free limited version of the Microsoft Visual Studio 2012 based on the Dot Net FrameWork 4.5 edition (https://visualstudio.microsoft.com/downloads/#d-express-windows-desktop). The program was written using the syntax roles of the VisualBasic.Net programing language. Using the designer part of Microsoft Visual Studio 2012 Express, the EgyGene GelAnalyzer4 main screens and its tools are adjusted: screen numbers of the program, screen sizes, fonts, and colors of every screen, drawing the tools on the screen for displaying the gel image and its detected lanes and bands, in addition to the tables for displaying the resulted data for the analysis of the gel image. On the other side, the coding part of the Microsoft Visual Studio 2012 Express software was used to write the whole instructions for the program EgyGene GelAnalyzer4 to work. VisualBasic.Net was used as a programing language to write the needed algorithms to do the whole program jobs: getting the color information of every pixel in the gel image, estimating the positions of lanes and bands, comparing the detected bands together to make the present/absent table, and using the present/absent table to calculate number of bands, gene and genotypic frequencies, polymorphism, and similarity percentages and draw phylogeny, in addition to the other image-relating tasks such as brightness and contrast adjustment.

### Icons editor

Icon is a small picture, with a square shape usually. The icon –as an image- consists of a grid of pixels with a specified width and height. The most common images have dimensions of 16 columns (icon width) and 16 rows (icon height) or simply 16 × 16 makes a grid of 256 pixels. By giving a specified color for every pixel, the icon is designed. To design the icons of EgyGene GelAnalyzer4 program, the freeware Greenfish Icon Editor Pro 3.2.5.1 (http://greenfishsoftware.org/gfie.php) was used to draw the icons. Most icons of the EgyGene GelAnalyzer4 program were squared moderately sized icons with 32 pixels width and 32 pixels height comprises a grid of 1024 individual pixels. The icons were designed using a transparent background to be more integrated with the background colors for the buttons of the EgyGene GelAnalyzer4 program that the user can change. The red color was the main used color to design the EgyGene GelAnalyzer4 icons with a light shadow of black and white to facilitate the icon visibility even the user chooses a red background for the EgyGene GelAnalyzer4 program buttons.

### Phylogenetic tree construction

The program constructs a phylogenetic tree using unweighted pair group method with arithmetic mean (UPGMA) method. Fourteen different equations [4, 14, 16, 18] are used to calculate the similarity percentages: Anderberg (*Sim*_*X, Y*_ *= a/[a + 2(b + c)]*), Czekanowski (*Sim*_*X, Y*_ *= 2a/[2a + b + c]*), Dice (*Sim*_*X, Y*_ *= 2a/[2a + b + c]*), Faith (*Sim*_*X, Y*_ *= [a + 0.5d]/[a + b + c + d]*), Gower and Legendre (*Sim*_*X, Y*_ *= [a + d]/[a + 0.5(b + c) + d]*), Jaccard (*Sim*_*X, Y*_ *= a/[a + b + c]*), Nei and Li (*Sim*_*X, Y*_ *= 2a/[(a + b) + (a + c)]*), Rogers and Tanimoto (*Sim*_*X, Y*_ *= [a + d]/[a + 2(b + c) + d]*), Russel and Rao (*Sim*_*X, Y*_ *= a/[a + b + c + d]*), simple matching (*Sim*_*X, Y*_ *= [a + d]/[a + b + c + d]*), Sokal and Michener (*[a + d]/[a + b + c + d]*), Sokal and Sneath 1 (*Sim*_*X, Y*_ *= a/[a + 2(b + c)]*), Sokal and Sneath 2 (*Sim*_*X, Y*_ *= 2(a + d)/[2(a + d) + b + c]*), and Sorensen (*Sim*_*X, Y*_ *= 2a/[2a + b + c]*). Where “*a”* means the number of shared bands between *X* and *Y* lanes, “*b*” is the number of bands displayed in *X* lane only, “*c*” is the number of bands represented by *Y* lane only and “*d*” is the number of bands which absent in both *X* and *Y* lanes.

## Results

### EgyGene GelAnalyzer history

EgyGene GelAnalyzer4 is the fourth version of the EgyGene GelAnalyzer software. The previous versions were released in 2004, 2005, and 2007, respectively. The fourth version provides many features that were not available in the previous three versions of the EgyGene GelAnalyzer. For example, the automatic detection of lanes and bands, splitting a lane into two lanes and merging two or more bands to be a single band. In addition, new five modules were not totally available in the first three versions: image enhancement, detection of molecular markers for some lanes, merging previous analyses together, drawing of phylogenetic tree, and calculation of population genetics parameters.

### The program main parts

**Fig. 1:**
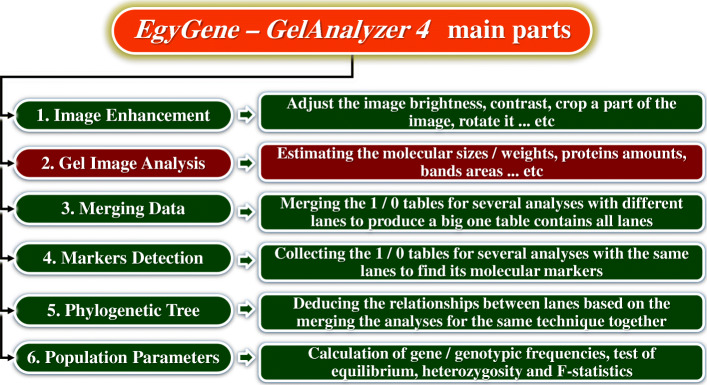
Illustration of the six main parts of the EgyGene GelAnalyzer4 software

The program consists of six main parts: gel image enhancement, image analysis, data merging, molecular markers detection, phylogenetic tree prediction, and population parameters estimation (Fig. 1).

#### Image enhancement part

The first part of the EgyGene GelAnalyzer4 software is the gel image enhancement section. It enhances the gel image colors and clarity. In this part of the program, researcher is able to adjust the image brightness and contrast, convert the colored image into a grayscale one, and reverse the image colors into their complemented ones. Also, a rotated image could be produced in a cure of the slippage of the gel at the image capturing stage. Flipping the image is available too in either horizontal or vertical manner. Cropping the image is an additional feature; user can crop the image from either outside or even inside. Inside cropping removes some inside lanes from the image. The last feature in the image enhancement section is a novel feature too: reorder lanes. Sometimes, samples are injected in the gel in a mis-order, and the reorder lanes feature treats this situation. The user determines the image lanes, then chooses the mis-ordered lane and moves it to its correct order, and the program cuts this lane and pastes it in the right user-defined place.

#### Image analysis part

##### Auto detection of lanes and bands

The second and the core part of the EgyGene GelAnalyzer4 program is the image analysis part. Once the image is entered to this part, the auto detection algorithm runs to detect lanes and bands, automatically. The auto detection algorithm gets the image pixels, processing it to discover the image lanes, searching in every lane for its bands, and drawing the detected lanes and bands on the image.

##### Lanes modifications

If the auto detection algorithm failed to detect the correct lanes in the image, user is able to modify the lanes by six different methods. The first way is to add new undefined lanes. The second method is to remove unreal defined lane(s). The third way is to resize the defined lane to fit the lane real borders. The fourth is to make a tilted lane with a variable length across their long. The other two methods are also novel features: lanes splitting and merging. With one click at the appropriate position in the lane, the single lane is split into two lanes. Lanes merging is the opposite process, which two or more lanes become a single one. By the way, if the defined lanes are changed, the auto detection algorithm runs automatically to redetect the bands again in the new lane.

##### Bands modifications

In a similar fashion to lanes modification, bands could be modified in five different ways: adding a new band, deleting an existing band(s), resizing detected band, and the two novel methods: splitting and merging bands.

##### Image analysis outputs

The results obtained by image analysis part in the EgyGene GelAnalyzer4 software includes (1) relative mobility, (2) molecular weights/sizes, (3) density (sums of the bright pixels), (4) DNA/protein quantity, (5) the presence/absence of each band across lanes, (6) the polymorphism, (7) the molecular markers for some selected lanes in the gel image, (8) phylogenetic tree for the samples in the analyzed images, (9) population parameters for the population of samples in the gel image and its sub-populations if there any sub-populations, and (10) the gel image with names of samples and marker values written on it.

#### Merging data

The third part of the program: data merging is a novel feature too. This part is for merging several pre-analysis to be one combined analysis. In many cases, the collected samples are larger than the maximum possible injected samples in one gel. In this case, the samples are injected into many gels; therefore, the same experiment has many images to be analyzed. The problem is, whatever the researcher unifies the experimental conditions; the migration of bands differs slightly between gels. So, to merge these data together you should compare the molecular weights/sizes of bands across all data/images to decide the matched bands together. The target of merging data part of the EgyGene GelAnalyzer4 software is to solve this problem. The program compares all the molecular weights/sizes of bands across all the entered data to match the bands across all the gels together. Two methods are available for merging the bands together: automatic and manual matching. In the automatic merging, the program uses a user-defined value as a maximum number of non-significant differences between bands to decide which bands are matched together. If the difference between the bands is less than the user-defined value, then all these bands are matched together as a same band with the average of their molecular weights/sizes as its new molecular weight/size. While in the procedure of manual matching, the user select the bands to merge them together whatever the values of differences between them. After finishing of bands matching, the rest bands considered as un-matched bands. In other words, these bands marked as unique bands for their gel/image. The results are displayed as one big table that represents the whole molecular weights/sizes and their presence/absence across all samples in all entered data/images/gels. This table is the input data in the other rest program parts: molecular markers detection, phylogenetic tree prediction, and population parameters estimation.

#### Molecular markers detection

The fourth section, molecular markers detection, is a novel feature too in gel analysis software. This program part uses data of several pre-analyzed images for the same samples to detect their molecular markers based on some user-defined criteria. Detection of molecular markers for the preferred group (positive markers) or for the un-preferred one (negative markers) is the target of this part. The input data of this part is the table of present/absent of bands. User can enter only one table (combined data from the previous part: merging data) or enter several tables for several experiments but with the same samples/genotypes. The program collects the entered data, making several sets of rows; every row set represents a particular image. The user just selects the individual samples for each group, then the program defines every positive or negative marker in it. The program saves the data of markers as well as the collected row sets; due to these row sets is the input data for the rest two parts of the program: phylogenetic tree prediction and population parameters estimation.

#### Phylogenetic tree construction

**Fig. 2:**
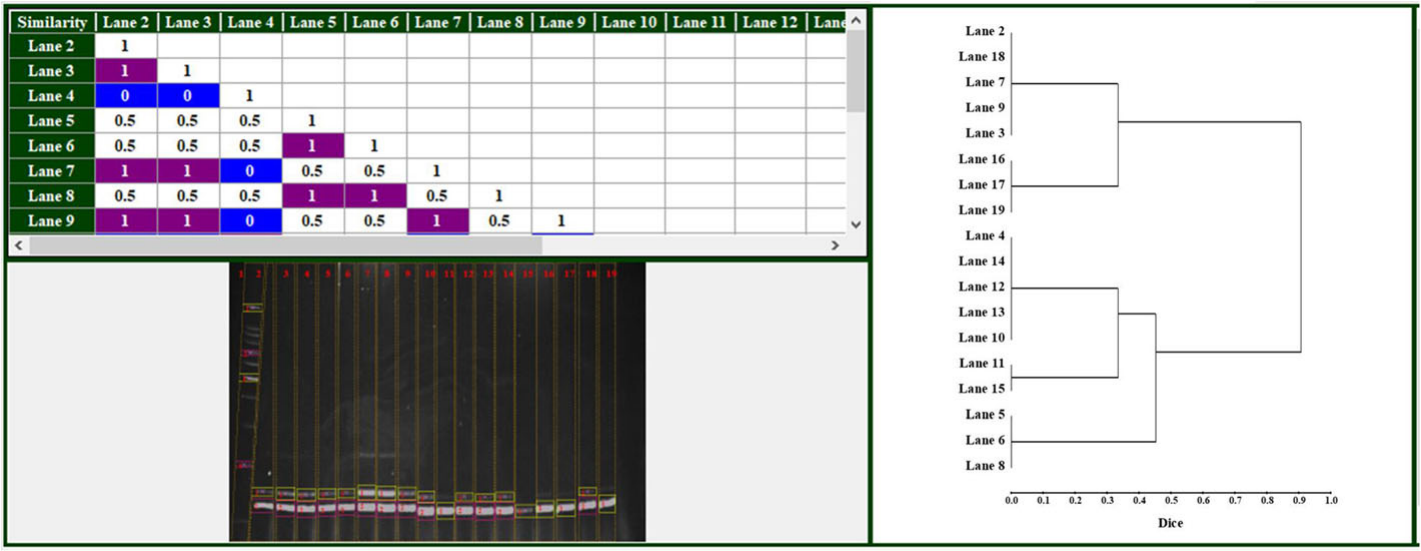
A part of the image analysis section of the program showing the analyzed image (bottom left), the phylogenetic tree (right), and the similarity percentages (top left) with blue and purple shading for the lowest and highest similarity percentages, respectively

The fifth part uses the outputs of the previous part: molecular markers detection to draw the phylogenetic tree between the image samples. The user can enter the pre-saved data in molecular markers detection or enter the gels one by one to collect them together to construct the phylogenetic tree. The user select the desired similarity equation (from the fourteen different similarity equations described previously) and the program calculate the similarity percentages and draw the phylogenetic tree. As many cases in the EgyGene GelAnalyzer4 program, the maximum and minimum values are colored to be easy to find (Fig. 2).

#### Population parameters

The last section calculates the population parameters for the pre-analyzed images before. The program calculates gene/allele and genotypic frequencies. Also, it estimates the observed and expected genotypic frequencies and numbers. It tests the Hardy-Weinberg equilibrium. It calculates the homozygosity and heterozygosity values and estimates the gene diversity. The program calculates the polymorphism information content too. If the image samples or the combined data is divided into sub-groups; the program calculates all the previous measures for the whole and each sub-population. In addition to the *F*-statistics for the sub-populations and the averages of all parameters for all entered loci.

## Discussion

### Ease of use

The program is designed to be simple, eye-attractive, and easy to use. The main displayed window contains six circles with numbers and the names of the six main parts of the program. If the user put the mouse cursor over any of them, the circle color is changed and a simple description of the function of this part (like those illustrated in Fig. 1) appears. Screens of the main six parts use the ribbon design; the icons have a flat appearance with transparent background to let the ribbon background to appear in the surrounding and also inside the icon; which makes a beautiful appearance. Also, if the user clicked any button, the background of the active button changes to give it a focus. The similarity percentages displayed in Fig. 2 have another two colors for distinguish the maximum and minimum similarity values to facilitate the easy finding of these values. This phenomenon is extended across all the six parts: whenever the user needs to distinguish between values, the program coloring the values for him. Of course, the user can change these all colors to be suited for him. Another phenomenon applied within the program is if any operation could be done without annoying the user; do it, do not ask him! For example, the operation of auto detecting lanes and bands is usually the first thing user does to start the image analysis; so, in the image analysis part, once the image is inserted, the program automatically shows the image to the user with its detected lanes and bands. This is on contrary with another powerful and easy-to-use gel image software such as GelAnalyzer2010a and GelJ which ask the user to detect the color of bands and gel background [7]. Keyboard shortcuts for all the operations in the program are provided for easy accessing the program commands.

### New outputs/features

EgyGene GelAnalyzer4 program resumes unfinished pre-saved analysis likes 78% of the other gel-image software [8]. It exports—like 78% of gel-image software—the output in the comma-separated format that recognized by spreadsheet programs like Microsoft Excel. This phenomenon makes the user able to perform further analyses/processing for the outputs [8], in addition to writing the names of samples and the detected molecular markers on the image as doing by 48% of the other competitor software [8]. However, EgyGene GelAnalyzer4 program provides some new outputs/features such as lanes reorder, splitting and merging of lanes and bands, and merging data and detection of molecular markers.

#### Lanes reorder

As mentioned above, in the image enhancement part, lanes reorder option is a new option in gel analysis software. If the lanes are injected in the gel in an incorrect order, EgyGene GelAnalyzer4 program cuts the lanes and re-paste them in a user defined order [8]. make a comparison study for the most available 33 commercial and freeware software for gel image analysis. In respect to the image enhancement section, they detected the six common/semi-common criteria to compare the 33 programs. Lanes reorder was not of them due to it is not addressed in the tested software. They found that 11 of 33 tested software did not include any image enhancement utility. Among the other 22 programs, about 17 of them allow image cropping and only about 7 programs allow the user to invert the image colors.

#### Splitting and merging of lanes and bands

EgyGene GelAnalyzer4 program offers the option for automatically detecting the lanes and bands. Also, the manual addition, deletion, and resizing of lanes and bands are included. In the comparison study of [8], about 84% of the tested 33 software offer the automatically/semi-automatically lanes/bands detection. About 84% of the software in [8] study determines unequal lane widths. The other 16% stalled with fixed width lanes. EgyGene GelAnalyzer4 program detects lanes with variable widths. Only 36% of the tested software in the study of [8] could deal with the curved lanes. EgyGene GelAnalyzer4 program provides tilted lanes feature, a similar approach to curved lanes. Both curved and tilted lanes solve the problem of un-straight lanes. Both the curved and tilted lanes have not a fixed width across their length. They may be wider/narrower in the top than in the lane bottom. The only difference between curved and tilted lanes is the tilted lane has straight not curved lines as those of the curved lane. In addition, EgyGene GelAnalyzer4 program provides novel features in the relation to detecting lanes and bands: splitting and merging. Splitting lane feature convert the bigger-than-reality lane/band into two lanes/bands in a single mouse click. If the algorithm of automatically detection of either lanes or bands makes a mistake by grouping two lanes (bands) into just one, the user can correct this mistake by deleting this big incorrect lane (band) and adding the correct two lanes (bands), or resizing it to fit only one of them and manually adding the other. However, in EgyGene GelAnalyzer4 software there is additional solution: splitting the big incorrect lane (band) by just one mouse click inside this lane (band). In addition, if the user faced the opposite situation, the same lane (band) detected as many (two or more) lanes (bands); EgyGene GelAnalyzer4 program provides the optimum solution of this problem by merging all the parted lanes (bands) together in the same correct one.

#### Merging data

In many experiments, number of samples exceeds the maximum number of samples of the gel electrophoresis apparatus. In this case, samples could not be electrophoresed together in the same gel. Few-to-many gels are used for the same experiment to cover the big number of samples. Different gels mean different conditions; which lead to variations in the migration of bands with the same molecular weights/sizes. Gel images are analyzed then their data are collected and compared together to elucidate the studied effects in the experiment. Merging the results of different gels together may be a difficult task especially in techniques such as microsatellites, which the bands may differ in just few bases. In this case, researcher can abstrusely distinguish between the differences between alleles and the differences between different gel conditions [12, 15]. EgyGene GelAnalyzer4 program deals with this problem using the merging data section; the program opens all the analyses results, collecting all the molecular weights/sizes across all samples in all images and comparing their values to matching the correct bands together as described previously.

#### Molecular markers detection

Molecular marker—in biology—is an expression for any stable heritable and detectable variation at the DNA level [5]. Molecular marker—on the gel image—is a polymorphic band that distinguishes between two groups of samples. Detection of molecular markers for specific samples helps plant breeders to improve plants by selecting the highly yielded genotypes [1]. Detection of molecular markers is a common task in identification and fingerprinting studies [1, 11]. Gel image software varies in their included features and its outputs [8]. Unfortunately, detecting the molecular markers in the analyzed image is not one of these features. Many software such as the powerful freeware *PyElph1.4* [10] compare the samples by its molecular weights/sizes to determine a one/zero table for the presence/absence of every band across all samples. These programs produce the presence/absence table but not provide the option of detecting the molecular markers may be due to its simplicity to do manually in a spreadsheet program such as Microsoft Excel. However, in a user view, it is bitter if this feature is already included in the gel image analysis [8]. EgyGene GelAnalyzer4 software provides this feature in three mouse clicks: two clicks for detecting the positive and negative samples, respectively and the third click is to enforce the program to define and show the detected molecular markers.

## Conclusion

EgyGene GelAnalyzer4 software provides some novel features such as lanes reorder in the gel image, splitting and merging of either lanes or bands, and merging gels data and molecular markers detection. In addition to the basic gel image analysis such as image enhancement, molecular weights/sizes calculation, drawing the phylogenetic tree, and more. This making EgyGene GelAnalyzer4 program a powerful software for gel image analysis.

## Data Availability

Not applicable
